# Case report: A therapeutic attempt to treat Morbihan disease with Baricitinib

**DOI:** 10.1016/j.heliyon.2024.e35547

**Published:** 2024-08-03

**Authors:** Luying Chen, Yanjuan Duan, Liang Zhao, Miao Xu, Yeqiang Liu, Xiaoxiang Zhai

**Affiliations:** a Department of Dermatology, Seventh People's Hospital of Shanghai University of Traditional Chinese Medicine, Shanghai, China; b Department of Dermatopathology, Shanghai Skin Disease Hospital of Tongji University School of Medicine, Shanghai, China

**Keywords:** Case report, Morbihan disease, JAK inhibitor, Baricitinib

## Abstract

A woman in her thirties who had been diagnosed with Morbihan disease did not notice a significant improvement in her condition after receiving years of treatment. Our decision to use Baricitinib helped her to achieve a better outcome. To our knowledge, this is the first study to use Baricitinib in Morbihan disease, although JAK inhibitors have already been successfully used before. It is hoped that our case report will provide new treatment options for Morbihan disease therapy.

## 1 Introduction

Morbihan disease, also known as Morbihan syndrome, is a rare skin disease that is characterised by chronic erythematous oedema in the upper and middle parts of the face. Currently, oral hormonal drugs, topical application of hormonal creams, calcineurin inhibitors, and laser therapy are common treatments for this condition. However, these treatments have poor efficacy, and better treatments are needed.

## 2 Case presentation

A 37-year-old woman who had been suffering from facial erythema and swelling for more than 5 years came to our hospital on September 30, 2022. The patient began to have redness and swelling on her face 5 years ago with no obvious cause and no self-reported symptoms. She was diagnosed with allergic dermatitis and rosacea and was treated with oral anti-allergy drugs, topical tacrolimus ointment, and redness-removing laser therapy. This treatment regimen had no apparent effect. Conversely, the redness and swelling gradually worsened, and the oedema of both upper eyelids was visible after rising in the morning. Later, she was given hydroxychloroquine and isotretinoin orally that helped slightly but the symptoms recurred, and the quality of her life was significantly impacted. Hence, she came to our department. The patient was in otherwise good health and had an unremarkable medical and family history with no drug or food allergies.

**Fig. 1:**
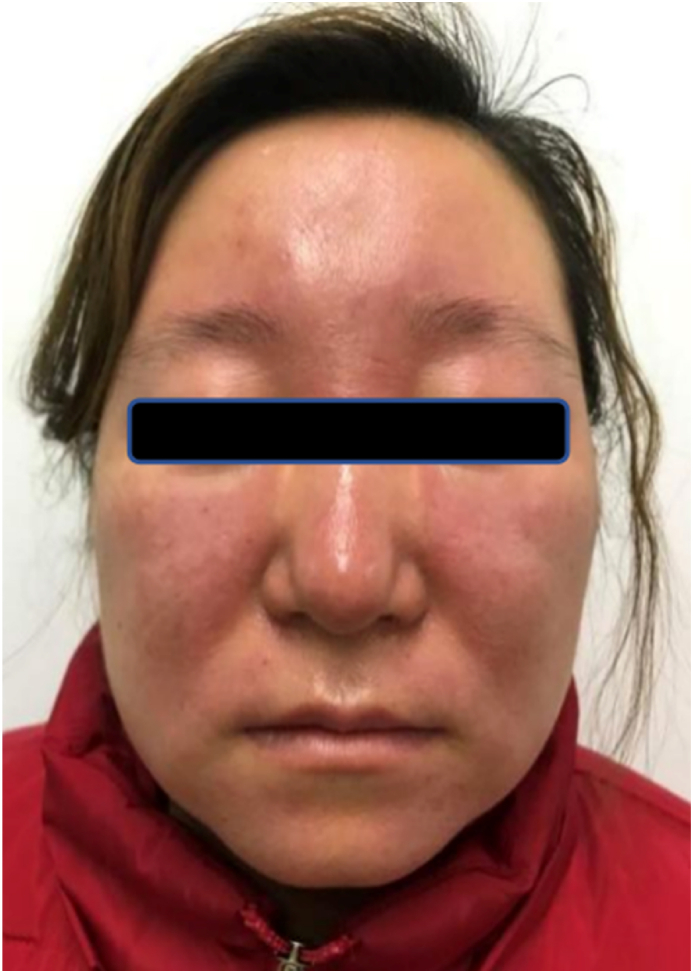
The pretreatment photo of prominent and persistent erythematous oedema of the face.

A physical examination of the patient revealed that she was in good general condition with no systemic abnormalities. A mild oedema of the frontal, periocular, cheek and nasal areas of the face, dark red complexion, indistinct lesion borders, infiltrative sensation on touch, normal skin temperature, and no pressure pain were noted (Fig. 1). We conducted more intrusive laboratory and auxiliary examinations on her. The results of all routine laboratory tests for blood and urine, C-reactive protein levels, thyroid function, liver and kidney function, and creatine myokinase levels were all normal. The results of tests for the presence of anti-nuclear antibody (ANA), anti-soluble extractable nuclear antigen (ENA) antibody, anti-double-stranded DNA antibody (ds-DNA), anti-Smith (Sm) antibody, and other autoantibodies were all negative. Meanwhile, blood concentrations of rheumatoid factor (RF), anti-streptococcal hemolysin O (anti-O), serum total complement (CH50), complement C3, and complement C4 were all normal. In addition, the results of allergen testing were normal, while the outcomes for fungal and trichinella testing were negative.

**Fig. 2:**
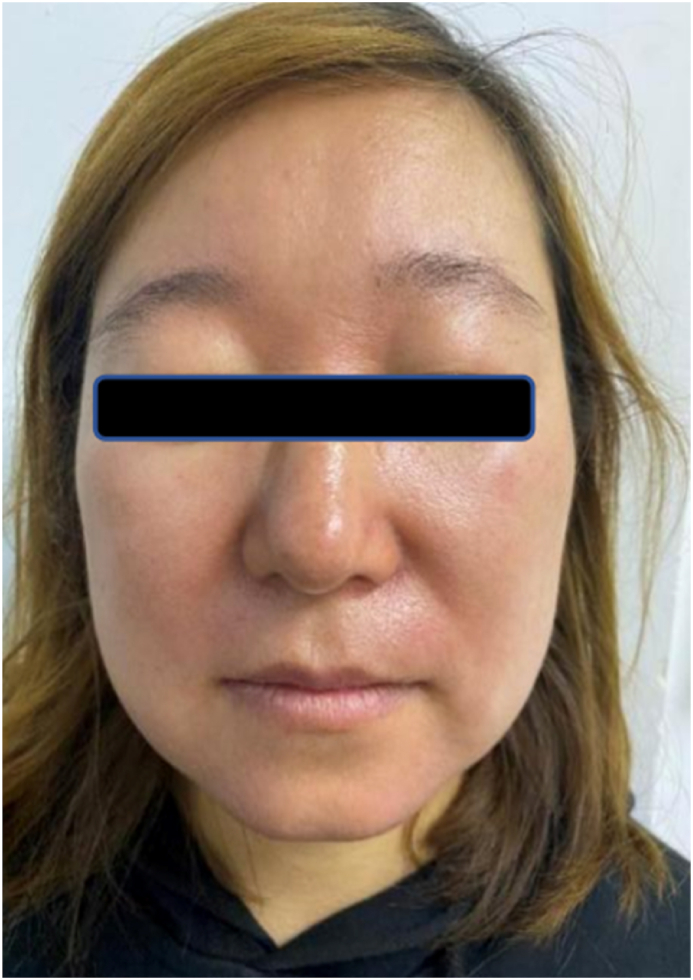
The patient was in her normal condition after daily oral Baricitinib for four weeks.

**Fig. 3:**
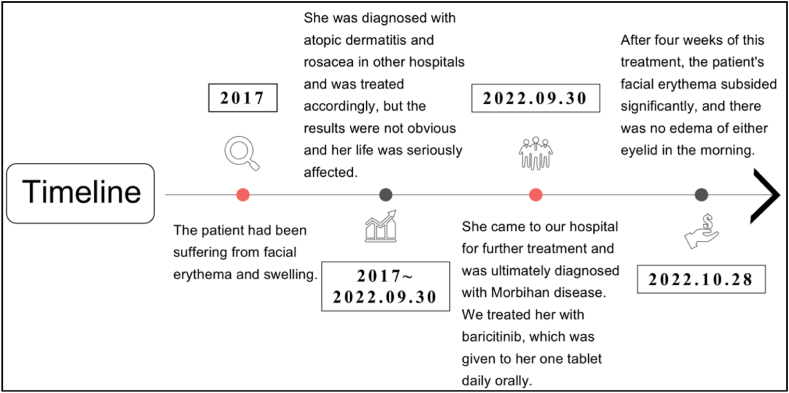
This is a timeline of the patient's condition from the beginning of their disease to the present.

In conclusion, the patient was definitively diagnosed with Morbihan disease. We treated her with Baricitinib that was administered orally at a dosage of one tablet of 2 mg daily. After 4 weeks of this treatment, the facial erythema of the patient subsided significantly and there was no oedema of both eyelids on waking up in the morning (Fig. 2). The patient felt that this was a simple and efficient course of treatment. She could also afford the medication.The dosage was not decreased for the entire four weeks that this patient was under treatment. Negative responses haven't been reported as of yet(Fig. 3).

## 3 Discussion

Morbihan disease, characterized by persistent erythema and solid swelling in the middle and upper two-thirds of the face, is clinically rare. It was first reported in 1957 by Dr. Degos, a French dermatologist, when describing a peasant patient in Morbihan, a region in northern France [1,2].

Morbihan disease primarily affects the forehead, cheek, nose, and periocular areas with nondepressed oedema appearing gradually that is accompanied by a flaky erythema with indistinct borders. Patients are predominantly middle-aged and elderly Caucasian males, and this condition is uncommon in Asian and Black populations [3,4]. Most patients have no significant self-reported symptoms, while some patients may present with lesions such as papules, pustules, nodules, and vasodilation [[5], [6], [7], [8]]. Persistent progressive erythematous oedema, particularly periorbital oedema, may affect facial appearance with significant facial disfigurement and visual field defects [9,10].

The etiology and pathogenesis of Morbihan disease remain unclear. It is speculated that the disease may be caused by inflammatory processes. Tissue biopsy shows localized lymphatic vessel oedema and loss of lymphatic vessel wall integrity that may be caused by chronic inflammation or other unknown factors (such as lymphatic dysfunction or other processes like physical tissue blockage due to infiltration or smooth muscle dysfunction) that lead to structural damage of blood vessels and lymphatic vessels, vascular dysfunction, and obstruction of lymphatic return, resulting in oedematous erythema [2,11,12]. Some studies have shown that Morbihan disease may be a rare complication of acne, while others have concluded that it is an unusual manifestation of rosace. However, most of the patients may not have acne or rosacea before or after the onset of the disease [3,13,14]. The cytokines produced by mast cells have been found to play a role in the pathophysiology of Morbihan disease in the recent case studies additionally.

Although Morbihan disease has specific clinical manifestations, there are no specific biochemical or other laboratory tests and no specificity in histopathology. Therefore, it is easily misdiagnosed clinically. It can be distinguishable from some diseases, such as facial myxedema, lupus erythematosus, dermatomyositis, allergic diseases, and others.

Morbihan disease may cause visual and facial appearance impairment due to lymphatic obstruction if it is not treated promptly. Although there is one report of Morbihan disease where the lesions disappeared spontaneously [15], the majority of patients have been reported to show a progressive development of the disease. Morbihan disease does not have a specific treatment because its etiology and pathogenesis are still unknown, it is refractory to treatment, and is a long-lasting condition. Some treatments have been reported, including both internal and external therapy. The internal treatments have included oral treatments with isotretinoin, doxycycline hydrochloride, prednisone acetate, tripterygium tablets, total glucosides of white paeony, and some antiallergic drugs, while the external treatments have included surgical therapies, topical application of hormonal creams, calcineurin inhibitors, metronidazole creams and intra-dermal injections of glucocorticoids. However, these treatments have only been reported for some individual cases or for a small group of patients. Moreover, each patient with Morbihan disease has a variable response to each treatment. There have also been a few reports of treatment resistance and recurrence of the disease after discontinuation of the drug [5,11,13,[16], [17], [18], [19], [20], [21], [22], [23], [24], [25]]. There also was a case report of the use of minocycline in combination with ketotifen in the treatment of Morbihan disease, and the patient has experienced rapid symptomatic relief after 7 days; the symptoms were completely resolved after 40 days of the drug, which showed the treatment was effective [26]. Slow-releasing doxycycline monohydrate has also been proposed as a kind of treatment for Morbihan's disease [27]. In addition, a case of Morbihan disease has been reported to be treated successfully with omalizumab in 2019 [28]. In conclusion, although there are numerous treatment options available, there is no clear indication of the probability of a positive response in a patient or any criteria for selecting a particular treatment for a patient.

The patient in our case was a 37-year-old woman with a 5-year long course of disease who had been systematically investigated for various diseases, with normal results for general blood biochemistry, immunological indicators, thyroid function, and other tests; the fungal and trichinella testing also gave negative results. In the early stages of the disease, our patient had been treated for dermatitis and rosacea with oral anti-allergy medication, topical tacrolimus ointment and laser treatment but the results were not positive. The disease continued to progress slowly, eventually leading to limited eye-opening ability in the morning. The patient was not examined via histopathological tests at the time of the first consultation in our hospital, and she was diagnosed with Morbihan disease on the basis of her clinical symptoms, treatment response, and literature review. According to the pre-treatment response, we chose to give the patient oral Baricitinib at a dosage of one tablet of 2mg daily. The erythema oedema of the patient subsided significantly after 28 days with no adverse effects.

The patient had been treated with several medications over the years; however, the results of treatment were not conclusive. Inflammatory processes may be one of the important pathogenic mechanisms of this disease. Zi-Yun Li published a case report about two Morbihan disease patients who had substantial improvement after receiving short-term Tofacitib treatment in 2023. Since Tofacitib is known to be a JAK inhibitor, the success of these two patients may also suggest that JAK inhibitors have a positive effect on Morbihan disease [29]. And given the good results of Janus-associated kinase (JAK) inhibitors in other difficult-to-treat diseases, we decided to select a JAK inhibitor for the treatment of Morbihan disease. JAK inhibitors can attenuate the local inflammatory response, modulate immunity, and ameliorate vascular dysfunction and lymphatic reflux disorders by inhibiting the JAK-STAT signalling pathway that may lead to an improvement in the facial erythema and oedema of the patient. Meanwhile, it is reported that Baricitinib has a lower incidence of adverse events, and a lower rate of drug resistance compared to Tofacitib. In addition, Baricitinib is cheaper than Abcixitinib that is a new generation JAK inhibitor. Considering all these reasons, we finally chose Baricitinib for the treatment of Morbihan disease.

Baricitinib is an orally available, specific inhibitor of JAKs (namely JAK1 and JAK2) that is approved for treating certain autoimmune and inflammatory disorders. It affects the transcription of DNA by influencing the JAK-STAT signalling pathway that is widely involved in many important biological processes such as cell proliferation, differentiation, apoptosis, immune regulation as well as in many inflammatory diseases. As a JAK inhibitor, Baricitinib is currently used in the treatment of rheumatoid arthritis. It has also been used to treat severe alopecia areata and moderate to severe atopic dermatitis [30]. It has a short half-life, acts on targeted critical pathways to reduce inflammation, and may have a beneficial therapeutic effect on patients with Morbihan disease.

## 4 Conclusion

The use of Baricitinib had a positive effect in this patient with Morbihan disease. This was probably related to the fact that Baricitinib inhibited inflammatory signalling pathways and improved the local inflammatory state. Based on our current review of the biomedical literature, this is the first report of the treatment of Morbihan disease with Baricitinib that provides an option and reference for similar interventions in the future. Nevertheless, this patient was treated for a short period of time, and the short-term efficacy was better than all the regimens used previously on her. The long-term efficacy of Baricitinib for treating Morbihan disease must be monitored continuously.

In addition, as this is a case report and the diagnosis of Morbihan disease is not an indication for administering Baricitinib, this result may have occurred by random chance and proper studies with a larger sample size are warranted in the future to confirm these findings. This case is intended to provide a new treatment option for the study of this disease and warrants further exploration in the future.

## Ethics statement

Written informed consent was obtained from the individual(s) for the publication of any potentially identifiable images or data included in this article.

## Funding

Innovative Team Projects of 10.13039/501100010032Shanghai Municipal Commission of Health (2022CX011); Young Qi-Huang Scholar of National Administration of Traditional Chinese Medicine; Training Program of the Seventh People's Hospital, 10.13039/501100010876Shanghai University of Traditional Chinese Medicine [QMX2021-05]; Characteristic Disciplines of Traditional Chinese Medicine (Traditional Chinese Medicine Dermatology) in Pudong New Area, Shanghai (YC-2023-0609); 10.13039/100014717National Natural Science Foundation of China [82004366]; Construction of famous Chinese medicine studios in Pudong [PDZY-2021-1009], The Featured Clinical Discipline Project of Shanghai Pudong (PWYts2021-16).

## Data availability

No data was used for the research described in the article.

## CRediT authorship contribution statement

**Luying Chen:** Writing – original draft. **Yanjuan Duan:** Methodology, Project administration. **Liang Zhao:** Conceptualization. **Miao Xu:** Data curation. **Yeqiang Liu:** Validation. **Xiaoxiang Zhai:** Writing – review & editing.

## Declaration of competing interest

The authors declare that they have no known competing financial interests or personal relationships that could have appeared to influence the work reported in this paper.
